# Inter-network connectivity and amyloid-beta linked to cognitive decline in preclinical Alzheimer’s disease: a longitudinal cohort study

**DOI:** 10.1186/s13195-018-0420-9

**Published:** 2018-08-28

**Authors:** Roy W. E. Van Hooren, Joost M. Riphagen, Heidi I. L. Jacobs, For the Alzheimer’s Disease Neuroimaging Initiative

**Affiliations:** 1 Faculty of Health, Medicine and Life Sciences; School for Mental Health and Neuroscience, Department of Psychiatry and Neuropsychology, Alzheimer Center Limburg, Maastricht University, Dr. Tanslaan 12, 6229 ET Maastricht, the Netherlands; 2 Department of Anesthesiology, Sankt-Willibrord Spital, Emmerich, Germany; 3 Faculty of Psychology and Neuroscience, Department of Cognitive Neuroscience, Maastricht University, Maastricht, The Netherlands; 4 Division of Nuclear Medicine and Molecular Imaging, Department of Radiology, Massachusetts General Hospital/Harvard Medical School, Boston, MA USA

**Keywords:** Alzheimer’s disease, Amyloid-β, Cognitively normal, Inter-network functional connectivity, Longitudinal, Memory performance, Mild cognitive impairment, Preclinical, Prodromal, Clinical trials

## Abstract

**Background:**

Amyloid-beta (Aβ) has a dose-response relationship with cognition in healthy adults. Additionally, the levels of functional connectivity within and between brain networks have been associated with cognitive performance in healthy adults. Aiming to explore potential synergistic effects, we investigated the relationship of inter-network functional connectivity, Aβ burden, and memory decline among healthy individuals and individuals with preclinical, prodromal, or clinical Alzheimer’s disease.

**Methods:**

In this longitudinal cohort study (ADNI2), participants (55–88 years) were followed for a maximum of 5 years. We included cognitively healthy participants and patients with mild cognitive impairment (with or without elevated Aβ) or Alzheimer’s disease. Associations between memory decline, Aβ burden, and connectivity between networks across the groups were investigated using linear and curvilinear mixed-effects models.

**Results:**

We found a synergistic relationships between inter-network functional connectivity and Aβ burden on memory decline. Dose-response relationships between Aβ and memory decline varied as a function of directionality of inter-network connectivity across groups. When inter-network correlations were negative, the curvilinear mixed-effects models revealed that higher Aβ burden was associated with greater memory decline in cognitively normal participants, but when inter-network correlations were positive, there was no association between the magnitude of Aβ burden and memory decline. Opposite patterns were observed in patients with mild cognitive impairment. Combining negative inter-network correlations with Aβ burden can reduce the required sample size by 88% for clinical trials aiming to slow down memory decline.

**Conclusions:**

The direction of inter-network connectivity provides additional information about Aβ burden on the rate of expected memory decline, especially in the preclinical phase. These results may be valuable for optimizing patient selection and decreasing study times to assess efficacy in clinical trials.

**Electronic supplementary material:**

The online version of this article (10.1186/s13195-018-0420-9) contains supplementary material, which is available to authorized users.

## Background

Elevated levels of amyloid-beta (Aβ), a neuropathological hallmark of Alzheimer’s disease (AD) [1–6], are crucial in identifying the earliest stages of AD. While Aβ does not relate well to cognition cross-sectionally, it has a dose-response relationship with cognition in healthy adults [7]. However, dementia-related pathologies, including Aβ, only explain 41% of variation in cognitive decline [8] and a low signal-to-noise ratio of biomarkers in the asymptomatic stages of the disease for entry criteria in clinical trials has been reported as a reason for trial failure [9].

Healthy aging has previously been associated with reduced activity within the default mode network (DMN), a network often associated with memory-related processes such as thinking about the future, episodic memory, and autobiographical memory [10]. Reduced connectivity within the DMN, as measured with resting-state functional magnetic resonance imaging, is also associated with impaired cognitive performance in old age [11]. Additionally, levels of functional connectivity within cognition-related intrinsic brain networks, such as the DMN, and Aβ burden have a synergistic effect on memory decline in clinically normal older individuals [12]. Various networks predict cognitive decline which may indicate that Aβ burden impacts the interaction between networks. Negative correlations between the DMN and task-positive networks has been positively associated with cognitive performance in young individuals [13, 14]. These negative correlation patterns are also referred to as “anti-correlations” in the literature and have been described as intrinsically organized antagonistic activation patterns between networks in the brain [15]. These patterns are reported to be part of a mechanism that facilitates cognition, possibly by reinforcing connections between two loci in the brain dedicated to cognitive functions [14, 16].

Previous studies have reported attenuated effects of aging with negative correlations between resting-state networks, with negative correlations further decreasing in patients with mild cognitive impairment (MCI) and AD [17–19]. While detrimental effects of Aβ on cognition and on functional connectivity within networks have been shown [2, 20–22], it remains unknown whether Aβ modulates functional connectivity between networks. Given the close relationship between inter-network connectivity and cognitive decline and these first reported associations with Aβ, combining information from inter-network connectivity with Aβ may reduce noise when selecting asymptomatic individuals at risk for AD in clinical trials aimed at slowing down cognitive decline.

To that end, we investigated whether functional connectivity between the DMN and task-positive networks predicts memory decline differently among cognitively normal individuals, MCI patients with and without elevated Aβ, or AD patients. Additionally, we investigated whether the relationship between inter-network connectivity and memory decline depends on Aβ levels in a dose-response type relationship. We expected that, as the magnitude of negative correlations decreases, memory performance would be lower, and that the strength of these associations would show a dose-response relationship with Aβ burden. To investigate domain-specificity of our findings, we have also investigated our hypotheses using executive functions as a control outcome measure.

## Methods

Data used in this article were obtained from the Alzheimer’s Disease Neuroimaging Initiative (ADNI) database (adni.loni.usc.edu). The ADNI was launched in 2003, led by Principal Investigator Michael W. Weiner, MD. The main goal of the ADNI is to test whether magnetic resonance imaging, positron emission tomography, other biological markers, and clinical and neuropsychological assessment can be combined to measure the progression of MCI and AD. For up-to-date information, see www.adni-info.org.

### Participants

We included data from a total of 122 eligible participants from the ADNI2 study (as of March 2017), of which seven were removed due to low imaging data quality, resulting in a total of 115 participants. Qualified clinicians working for the ADNI categorized participants into four groups (cognitively normal, early MCI, late MCI, or AD) based on diagnostic procedures from the ADNI protocol [23]. However, for the purposes of our study, we grouped all patients with MCI based on their Aβ levels being below or above a pathological cutoff point of 1.11 ^18^F-AV-45 florbetapir positron emission tomography standardized uptake value ratio [24], respectively (referred to as MCI^–^ and MCI^+^ in this manuscript). MCI diagnoses were based on the Petersen criteria for MCI [25, 26], while patients with AD met the NINCDS-ADRDA criteria for probable Alzheimer’s disease [27]. For a detailed overview of exclusion, inclusion, and diagnostic criteria and procedures, please refer to the ADNI2 protocol (http://adni.loni.usc.edu/wp-content/uploads/2008/07/adni2-procedures-manual.pdf). Additionally, only participants with complete resting-state functional magnetic resonance imaging and Aβ data at baseline were eligible for inclusion, and patients with AD were only eligible if their Aβ levels were above the cutoff point to ensure only AD-related pathology was represented in this group. A complete listing of all participant IDs that were included in the final analyses can be found in Additional file 1 (Table S1).

### Materials and equipment

#### Test batteries

The main outcome measures we used were the composite memory score (ADNI-Mem) and the composite executive functions score (ADNI-EF), which have good validity and are ideally suited to track changes over time. These scores were derived by combining scores related to memory performance and executive functions. ADNI-Mem score is derived from several test batteries, including the Rey Auditory Verbal Learning Test, Alzheimer’s Disease Assessment Schedule-Cognition, Mini-Mental State Examination, and Wechsler Memory Scale-Revised [28]. ADNI-EF score is derived from tests including WAIS-R Digit Symbol Substitution, Digit Span Backwards, Trails A and B, Category Fluency, and Clock Drawing [29].

#### Biomarker assessment

^18^F-AV-45 florbetapir positron emission tomography measures were used to quantify levels of neocortical Aβ at baseline. The duration of positron emission tomography imaging was 20 min and started 50 min after injection of tracer fluid. The neocortical standardized uptake value ratio is the mean uptake in an aggregate of the frontal lobe, cingulate cortex, lateral parietal, and lateral temporal regions relative to mean uptake in the whole cerebellum, including white and gray matter. Further processing of positron emission tomography images was performed as described in a previous report [24]. Participants were characterized as Aβ-positive if they exceeded the cutoff value of 1.11 standardized uptake value ratio, as previously determined in ADNI cohorts [24].

#### Imaging equipment and acquisition

Imaging data were acquired using Philips Medical Systems 3.0-Tesla magnetic resonance systems. Structural T1-weighted gradient echo pulse sequence data with dimensions of 170 × 256 × 256 mm with a voxel resolution of 1.2 × 1 × 1 mm were acquired in sagittal orientation with a repetition time of 6.8 ms, echo time of 3.1 ms, flip angle of 9°, and slice thickness of 1.2 mm. Additionally, resting-state functional magnetic resonance imaging scans of 7 min were obtained, consisting of 140 volumes of T2*-weighted data with 48 slices per volume, dimensions of 64 × 64 × 48 mm, and a voxel resolution of 3.3 × 3.3 × 3.3 mm. Functional images were acquired in transverse orientation with repetition time of 3000 ms, echo time of 30 ms, flip angle of 80°, and a slice thickness of 3.3 mm.

#### Image preprocessing and de-noising

For preprocessing we used the default preprocessing pipeline for volume-based analyses within the NITRC CONN toolbox (version 17.a; http://www.nitrc.org/projects/conn/) [30]. Images were realigned and unwarped, centered, slice-time corrected, segmented (gray/white/cerebrospinal fluid), normalized to MNI space, and outliers were identified with the Artifact Detection Toolbox. Outliers were regressed out (scrubbing) using conservative settings (95th percentile in normative sample, global signal Z value = 2, motion = 0.5 mm, and we discarded the first three volumes). Functional data were smoothed with a Gaussian kernel of 6 mm full width at half maximum. As the reliability and validity of negative correlation measures has been the subject of debate [31], de-noising of data involved regressing out principal components of the signal from white matter and cerebrospinal fluid following the CompCor method [32] which reduces the presence of artificial negative correlations. Additionally, linear detrending and an after-regression BOLD signal band-pass filter (0.008 < f <  0.09 Hz) were applied. Data quality was ensured by visual inspection of histograms of functional connectivity values before and after de-noising for each participant. Histograms that did not show a normal distribution of functional connectivity values after de-noising were indicative of a suboptimal de-noising process and these participants were excluded from data analysis to preserve a high level of data quality (*n* = 7). Motion parameters were entered as regressor in our analyses. Volumes with motion above 0.5 mm were entered as regressors in our general linear models (“scrubbing”). Furthermore, all images were inspected for irregularities during the entire preprocessing and analysis process.

#### Functional connectivity analyses

**Fig. 1:**
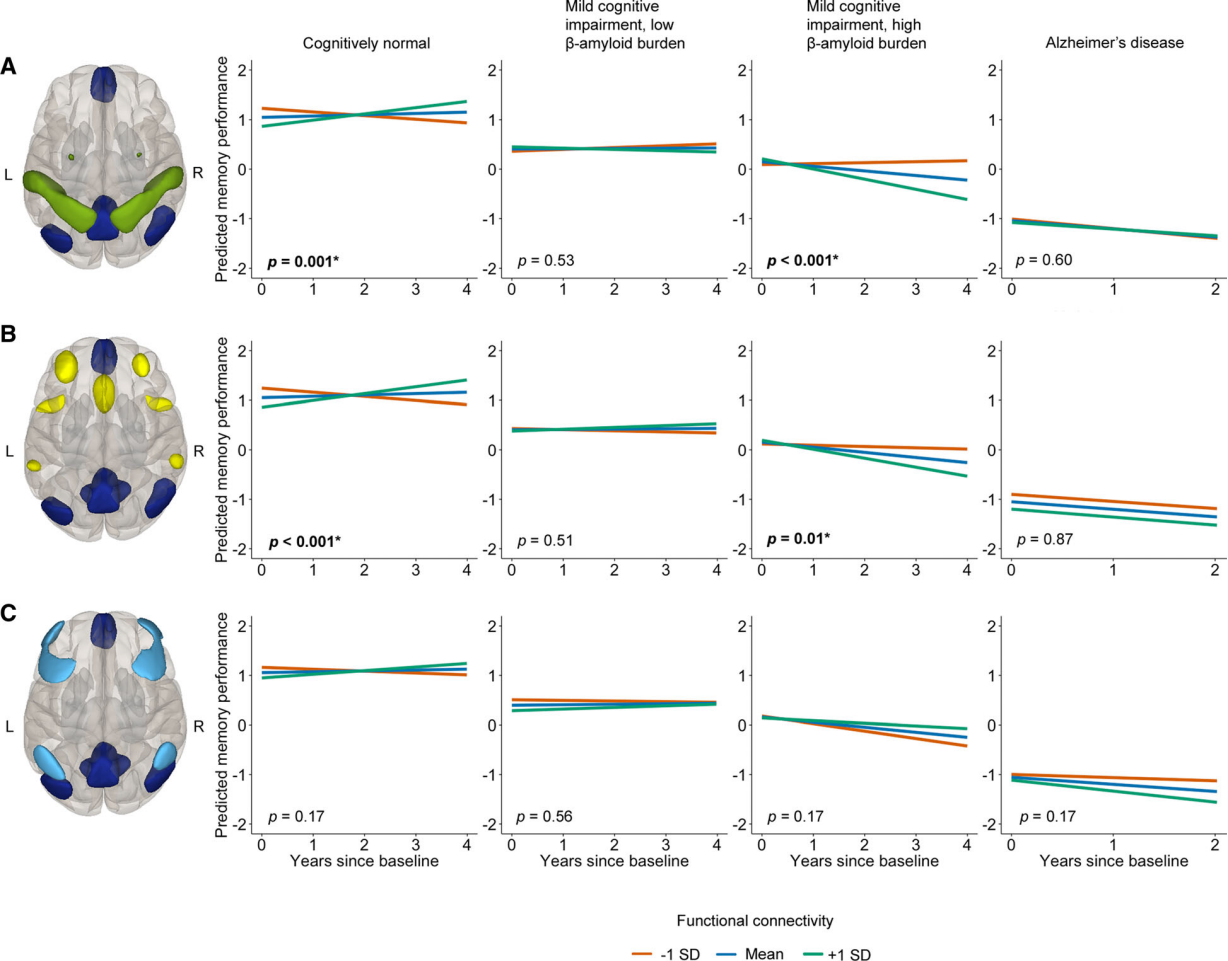
Linear effects of functional connectivity on memory decline over time per group. The effect of baseline functional connectivity between networks on memory performance over time was investigated in three network pairs in all groups. Functional connectivity values were standardized to ensure that the mean reflects a z-value of 0. Red, green, and blue lines indicate the estimated marginal means for the moderation by negative (–1 standard deviation (SD)), positive (+1 SD), and no (mean) correlation between networks, respectively, but the analyses were performed using functional connectivity measures continuously. Network combinations from top to bottom: DMN-DAN, DMN-SN, and DMN-FPN. The DMN is shown in dark blue, the DAN in green, the SN in yellow, and the FPN in light blue. The brain images give a superior viewpoint of the brain; top = anterior, L = left, R = right, bottom = posterior. The AD group only had a maximum follow-up time of 2 years (Table 1). All *p* values are corrected for multiple comparisons using FDR. Significant effects are indicated by an asterisk and bold font. **a** DMN-DAN: significant effects were found in the cognitively normal and MCI^+^ groups. **b** DMN-SN: significant effects were found in the cognitively normal and MCI^+^ groups. **c** DMN-FPN: no significant effects were found for this network pair

A bivariate correlation, hemodynamic response function-weighted ROI-to-ROI analysis was performed within CONN, using four independent network regions of interest (ROI) as sources, with a total of 19 structurally defined hub regions. The regions of interest include the DMN and regions representing task-positive networks, including the dorsal attention network (DAN), salience network (SN), and frontoparietal network (FPN). These regions of interest, which are part of the CONN software package, were generated by an independent component analysis on 497 healthy control participants (293 females) as part of the Human Connectome Project (http://www.humanconnectome.org) [30]. As a reference for future replication efforts, peak coordinates of the regions of interest used for this functional connectivity analysis are provided in Additional file 1 (Table S2) and the networks used are visualized in Fig. 1. To assess inter-network connectivity, the average time course signal from each network was extracted and Fisher r-to-z transformed inter-network correlation values were exported for statistical analyses.

### Statistical analysis

All data were analyzed using R version 3.3.1 (https://www.r-project.org/) and MATLAB R2016a. Baseline participant characteristics were compared with analysis of variance for continuous variables and chi-squared test for categorical variables. Linear regression analyses were used for cross-sectional data. Because of their ability to deal with missing data in longitudinal studies without being subject to complete-case bias [33, 34], longitudinal analyses were performed with linear and curvilinear mixed-effect regression analyses using maximum likelihood, utilizing nlme version 3.1–128 [35]. Fixed effects were predictor of interest, a random intercept for each participant, and random slope for time. Time was calculated as the number of years since baseline assessment. During the construction of statistical models, we first investigated effects in the whole sample. In case of a significant effect, the sample was split based on clinical groups and the level of amyloid burden, for which interactive analyses were performed with the cognitively normal group as reference. We also performed within-group analyses to better understand the patterns. For all models, we compared the Akaike Information Criteria between models with a random intercept and random slope or a random intercept only using the Log-likelihood ratio test and selected the most parsimonious model. In all models, age, sex, education, and their interaction with time were included as covariates if *p* <  0*.*10 (using the Wald *t* statistic) [36]. To improve longitudinal data quality [37], variability in head size and intracranial volume, as measured with FreeSurfer version 5.1 [38], was added as a covariate [39]. Additionally, intracranial volume can be used as a measure of head size, which correlates with total gray matter atrophy. It is important to control for atrophy as atrophy can reduce blood flow [40], and AD patients may show more atrophy than patients with MCI or healthy controls. The most complex model constructed for analyses is described in detail in Additional file 1 (Box 1). Residual plots and Q-Q plots were examined for all models. Significance was set at *p* <  0*.*050 (two-sided) and results were corrected for multiple comparisons using the false-discovery rate (FDR). Power calculations were performed using MATLAB scripts for the mixed-effects model using the slope of memory decline and residual variance [40] to estimate the number of participants a clinical trial would need to enroll to detect slowing of memory decline of 30% (two arms for 4-year annual assessments, 80% power, alpha = 0.05) using high amyloid levels as an inclusion criterion. These mixed-effects models included the covariates, random intercept, and random slope.

## Results

### Demographics

The total sample consisted of 115 participants, including 53 females (46%) with a mean age of 72.91 (standard deviation (SD) = 6.68) at baseline. Of these participants, 28 were in the cognitively normal group, 27 were in the MCI^–^ group, 36 were in the MCI^+^ group, and 24 were in the AD group. Other participant characteristics are summarized in Table 1.

**Table 1:** Summary table of participant characteristics at baseline

Variable	CN (*n* = 28)	MCI^–^ (*n* = 27)	MCI^+^ (*n* = 36)	AD (*n* = 24)	*p* value
AV45 SUVR	1.16 (0.21)	1.01 (0.05)	1.39 (0.17)	1.50 (0.15)	< 0.001
Age (years)	74 (5.71)	71.36 (8.03)	72.58 (5.79)	74 (7.32)	0.42
Education (years)	16.43 (1.93)	16.48 (2.68)	15.86 (2.46)	15.46 (2.50)	0.36
Females	15 (54%)	12 (44%)	14 (39%)	12 (50%)	0.67
ICV (mm^3^)	1,572,034 (153,316.20)	1,545,103 (168,923.10)	1,568,839 (174,101.60)	1,574,218 (210,862)	0.94
APOE ε4	10 (36%)	4 (15%)	24 (66%)	20 (83%)	< 0.001^a^
ADNI-Mem	0.89 (0.52)	0.37 (0.49)	0.15 (0.60)	−1.10 (0.56)	< 0.001
ADNI-EF	0.80 (0.56)	0.25 (0.80)	0.34 (0.86)	−0.94 (0.62)	< 0.001
CDR-SB	0.05 (0.16)	1.26 (0.92)	1.90 (1.06)	4.41 (1.31)	< 0.001
FC DMN-DAN	−0.12 (0.28)	0.00 (0.33)	−0.03 (0.24)	−0.15 (0.30)	0.17
FC DMN-SN	−0.15 (0.29)	−0.11 (0.28)	−0.08 (0.28)	−0.18 (0.27)	0.56
FC DMN-FPN	0.31 (0.23)	0.20 (0.30)	0.26 (0.23)	0.17 (0.29)	0.24
Follow-up time (years)	1.63 (1.24)	1.30 (0.96)	1.46 (1.09)	0.90 (0.53)	0.001

Data are presented as the mean (standard deviation) for the continuous variables and as *n* (%) for the categorical variable

The APOE ε4 row gives the number of participants in this group with one or more APOE ε4 alleles

Statistical significance was tested with analysis of variance for continuous variables and chi-squared test for the categorical variables

*APOE ε4* apolipoprotein ε4, *AD* Alzheimer’s disease, *ADNI-EF* Alzheimer’s Disease Neuroimaging Initiative executive functions score composite, *ADNI-Mem* Alzheimer’s Disease Neuroimaging Initiative memory score composite, *AV45* ^18^F-AV-45 florbetapir, *CDR-SB* Clinical Dementia Rating scale—sum of boxes, *CN* cognitively normal, *DAN* dorsal attention network, *DMN* default mode network, *FC* functional connectivity, *FPN* frontoparietal network, *ICV* intracranial volume, *MCI* mild cognitive impairment, *SN* salience network, *SUVR* standardized uptake value ratio

^a^ All groups were significantly different from each other, except CN versus MCI^–^ and MCI^+^ versus AD

Analyses of variance indicated group differences in ^18^F-AV-45 florbetapir standardized uptake value ratio, ADNI-Mem, ADNI-EF, clinical dementia rating scale scores, and follow-up times. The proportion of cases at each time point were as follows: baseline, 115 (100%); month 6, 110 (96%); month 12, 98 (85%); month 24, 74 (64%); month 36, 6 (5%); month 48, 29 (25%); and month 60, 1 (< 1%). Results of Tukey tests for post-hoc differences between group characteristics are available in Additional file 1 (Table S3). Additionally, we found no differences in image preprocessing parameters between groups (Additional file 1: Table S4).

### Effects of functional connectivity and group on memory at baseline

There were no significant associations between functional connectivity between networks and memory performance at baseline across the entire sample or within the groups. An association was found between functional connectivity between the DMN and FPN and executive functions across the entire sample at baseline. Further analyses of the association between functional connectivity and executive functions within the diagnosis groups reveal an association in the amyloid-negative MCI group (Additional file 1: Table S5).

### Longitudinal linear effects of functional connectivity and group on memory

Linear mixed-effect models were performed to investigate effects of functional connectivity and group on memory decline over time. The associations between functional connectivity between the networks and time for the whole sample were not significant (Additional file 1: Table S6). Using the cognitively normal group as a reference level, a significant three-way interaction effect of time, group, and functional connectivity was found in comparison with the MCI^+^ group for the DMN-DAN and DMN-SN correlations, but not for the DMN-FPN correlations (Table 2). Adding inter-network functional connectivity to the models with the covariates contributed significantly to the explained variance of memory decline for the DMN-DAN (*R*^2^ difference = 0.03, 95% confidence interval (CI) 0.01 to 0.07; *p* = 0.04), but not for the DMN-SN (*R*^2^ difference = 0.001, 95% CI −0.001 to 0.01; *p* = 0.21) or the DMN-FPN (*R*^2^ difference < 0.001, 95% CI −0.001 to 0.01; *p* = 0.48).

**Table 2:** Associations between group, inter-network functional connectivity, and memory decline

Fitted model	Est.	SE	95% CI	DF	T value	*p* value	*p* value (FDR)
DMN-DAN (*n* = 115, number of observations = 433)							
Time × MCI^–^ × DMN-DAN	−0.43	0.22	−0.68 to −0.01	310	−2.00	0.047	0.14
Time × MCI^+^ × DMN-DAN	−0.79	0.23	−1.25 to −0.34	310	−3.43	0.001	0.001
Time × AD × DMN-DAN	−0.24	0.30	−0.84 to 0.36	310	−0.79	0.43	0.43
DMN-SN (*n* = 115, number of observations = 433)							
Time × MCI^–^ × DMN-SN	−0.26	0.23	−0.71 to 0.19	310	−1.13	0.26	0.32
Time × MCI^+^ × DMN-SN	−0.55	0.20	−0.93 to −0.16	310	−2.81	0.01	0.01
Time × AD × DMN-SN	−0.36	0.31	−0.98 to 0.25	310	−1.17	0.25	0.37
DMN-FPN (*n* = 115, number of observations = 433)							
Time × MCI^–^ × DMN-FPN	−0.27	0.27	−0.81 to 0.26	310	−1.00	0.32	0.32
Time × MCI^+^ × DMN-FPN	−0.21	0.26	−0.72 to 0.30	310	−0.81	0.42	0.42
Time × AD × DMN-FPN	−0.45	0.33	−1.09 to 0.19	310	−1.38	0.17	0.37

Results are acquired using the cognitively normal group as a reference group

Regression models are adjusted for age, intracranial volume, sex, and education

Beta coefficients are unstandardized

*AD* Alzheimer’s disease, *CI* confidence interval, *DAN* dorsal attention network, *DF* degrees of freedom, *DMN* default mode network, *FDR* false discovery rate, *FPN* frontoparietal network, *MCI* mild cognitive impairment, *SE* standard error, *SN* salience network

Figure 1 and Table 2 show significant associations between DMN-DAN and DMN-SN functional connectivity and memory decline between the cognitively normal and MCI^+^ groups. Post-hoc linear mixed models within each group confirmed that, for the cognitively normal group, positive correlations between these network pairs are positively associated with memory performance over time, whereas in the MCI^+^ group positive correlations were negatively associated with memory performance (Additional file 1: Table S7). No significant associations were found for executive functions (Additional file 1: Table S8).

As the cognitively normal group consisted of 11 individuals with Aβ levels above the threshold, we also examined associations between functional inter-network connectivity, Aβ as a continuous variable, and memory decline in the cognitively normal group. These results showed that this interaction was significant for the DMN-DAN and the DMN-SN, but not for the DMN-FPN. This shows that Aβ moderates the association between functional connectivity and memory decline also in the cognitively normal group in such a way that negative correlations were associated with memory decline with elevated Aβ (Table 3). Similar findings are observed when grouping cognitively normal individuals based on the Aβ cutoff value (Additional file 1: Table S9).

**Table 3:** Three-way interaction of functional connectivity, amyloid-beta, and time in the cognitively normal group

Fitted model	Est.	SE	95% CI	DF	T value	*p* value	*p* value (FDR)
DMN-DAN (*n* = 28, number of observations = 118)							
DMN-DAN × Aβ × Time	1.97	0.48	1.03 to 2.92	86	4.14	< 0.001	< 0.001
DMN-SN (*n* = 28, number of observations = 118)							
DMN-SN × Aβ × Time	1.86	0.56	0.74 to 2.97	86	3.31	0.001	0.001
DMN-FPN (*n* = 28, number of observations = 118)							
DMN-FPN × Aβ × Time	−0.62	0.97	−2.55 to 1.30	86	−0.64	0.52	0.52

Regression models are adjusted for age, intracranial volume, sex and education

Beta coefficients are unstandardized

*Aβ* amyloid-beta, *CI* confidence interval, *DAN* dorsal attention network, *DF* degrees of freedom, *DMN* default mode network, *FPN* frontoparietal network, *SE* standard error, *SN* salience network

### Longitudinal curvilinear effects of functional connectivity and amyloid-beta on memory

**Fig. 2:**
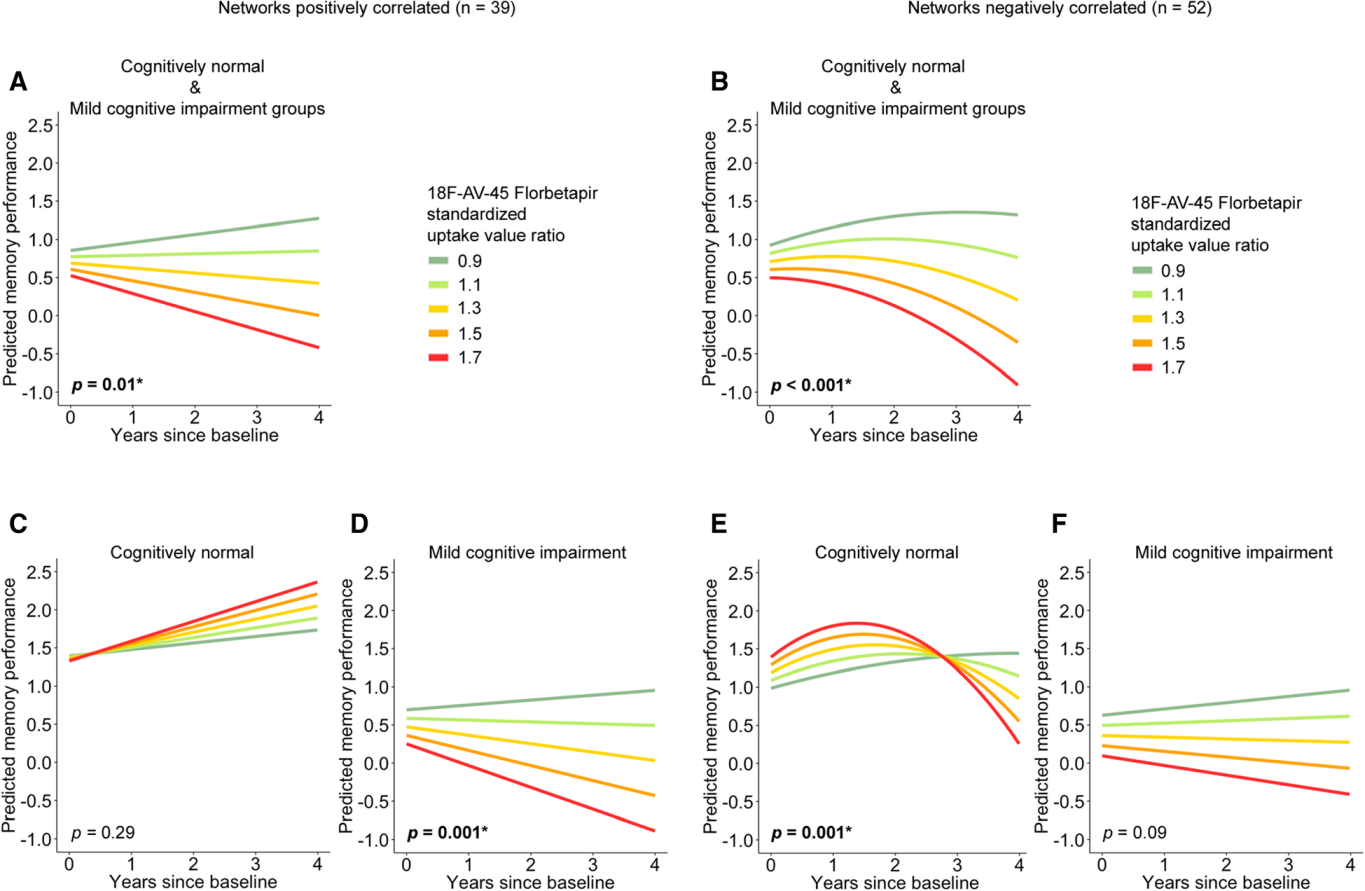
Curvilinear three-way interaction of functional connectivity, amyloid-beta and time and its relationship with memory performance. To visualize the three-way interaction, the whole sample, excluding the AD group, was split based on whether functional connectivity between DMN-DAN was below or above zero, allowing us to visualize the dose-response relationship of Aβ burden (measured with ^18^F-AV-45 florbetapir standardized uptake value ratio) on memory decline in both subgroups. ^18^F-AV-45 florbetapir standardized uptake value ratio values over 1.11 indicate Aβ positivity. Linear or curvilinear graphs were drawn based on which type of association showed the best fit for each model. All *p* values are FDR-corrected. Significant effects are indicated by an asterisk and bold font. **a** Effects within the subgroup of cognitively normal participants and MCI patients showing positive correlations between networks. **b** Curvilinear effects within the subgroup of cognitively normal participants and MCI patients showing negative correlations between networks. **c** Effects within the cognitively normal subgroup with positive correlations between networks; no significant dose-response relationship of Aβ on memory (practice effects). **d** Effects within the MCI subgroup with positive correlations between networks showing a dose-response relationship of Aβ on memory. **e** Effects within the cognitively normal subgroup with negative correlations between networks showing a curvilinear dose-response relationship of Aβ on memory. **f** Effects within the MCI subgroup with negative correlations between networks showing no (borderline) significant effect of Aβ burden on memory

Since our linear mixed model results showed opposite moderations by Aβ on the association between functional connectivity and memory performance across the diagnostic groups, we examined curvilinear (quadratic) mixed-effect models for these network interactions using Aβ as a continuous variable to investigate a possible dose-response relationship with memory decline. To ensure that individuals with the highest Aβ levels would not drive associations, we excluded the AD group from these analyses. In the whole sample, excluding the AD group, a quadratic three-way interaction effect of functional connectivity, Aβ, and time was found for the DMN-DAN, but marginally not for the DMN-SN correlations (Table 4). To visualize this interaction, we plotted simple slopes for different values of ^18^F-AV-45 florbetapir standardized uptake value ratio in both groups, broken down by positive and negative inter-network correlations and holding all other fixed effects constant (Fig. 2). Values for Aβ were chosen to reflect Aβ negativity (0.9), the cutoff (1.1), slightly elevated Aβ (1.3), moderately elevated Aβ (1.5), and high Aβ (1.7).

**Table 4:** Curvilinear associations between functional inter-network connectivity, amyloid-beta burden, and memory decline

Fitted model	Est.	SE	95% CI	DF	T value	*p* value	*p* value (FDR)
Whole sample, excluding AD group (*n* = 91, number of observations = 364)							
DMN-DAN (*n* = 91, number of observations = 364)							
DMN-DAN × Aβ × (Time^2^)	0.42	0.16	0.10 to 0.74	266	2.60	0.01	0.02
DMN-SN (*n* = 91, number of observations = 364)							
DMN-SN × Aβ × (Time^2^)	0.25	0.14	−0.02 to 0.52	266	1.84	0.07	0.07
Subset of participants with positive correlation between DMN and DAN^b^							
Whole sample, excluding AD group (*n* = 39, number of observations = 157)							
Aβ × Time	−0.43	0.15	−0.73 to −0.13	116	−2.83	0.01	0.01
CN (*n* = 10, number of observations = 43)							
Aβ × Time	0.22	0.20	−0.19 to 0.63	31	1.08	0.29	0.29
MCI (*n* = 29, number of observations = 114)							
Aβ × Time	−0.44	0.13	−0.69 to −0.18	83	−3.43	0.001	0.001
Subset of participants with negative correlation between DMN and DAN^a^							
Whole sample. Excluding AD group (*n* = 52, number of observations = 207)							
Aβ × (Time^2^)	−0.05	0.01	−0.07 to − 0.03	152	−4.23	< 0.001	< 0.001
CN (*n* = 18, number of observations = 75)							
Aβ × (Time^2^)	−0.25	0.08	−0.41 to − 0.10	53	−3.33	0.001	0.001
MCI (*n* = 34, number of observations = 132)							
Aβ × Time	− 0.26	0.15	− 0.56 to 0.04	96	−1.73	0.09	0.09

Regression models are adjusted for age, intracranial volume, sex and education

Beta coefficients are unstandardized

*Aβ* amyloid-beta, *AD* Alzheimer’s disease, *CI* confidence interval, *CN* cognitively normal, *DAN* dorsal attention network, *DF* degrees of freedom, *DMN* default mode network, *FDR* false discovery rate, *MCI* mild cognitive impairment, *SE* standard error, *SN* salience network

^a^ Models include a quadratic term only if it explained more variance than the linear model without quadratic term

Figure 2 shows that the dose-response relationship between Aβ and memory decline is modulated by both diagnosis and the direction of DMN-DAN correlations. Within the cognitively normal group, negative DMN-DAN correlations have a curvilinear dose-response relationship with memory decline. However, when networks are positively correlated, we found no significant association between Aβ and memory decline. Conversely, in the MCI group, positive DMN-DAN correlations were associated with a linear dose-response relationship between Aβ and rate of memory. No moderation effects of Aβ were found for the MCI group with negative correlations, although linear effects approached significance (Table 4).

**Fig. 3:**
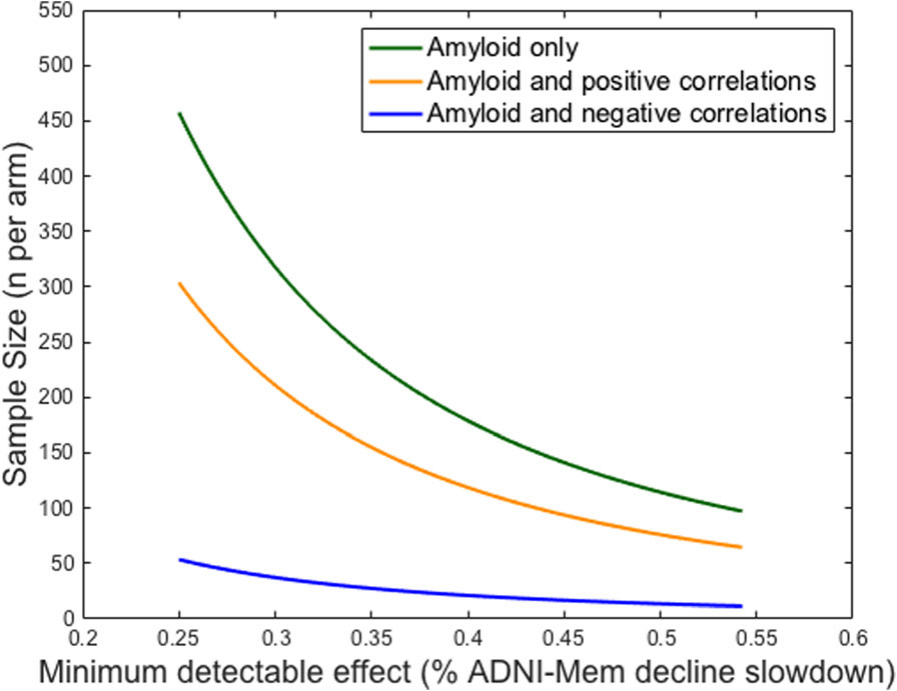
Power analysis in the cognitively normal group. To assess the effect of including inter-network connectivity as an inclusion criterion in clinical trials, we performed a power analysis in the cognitively normal group. The *x* axis describes the memory slope reduction in percentages and the *y* axis describes the number of participants needed per arm to detect this reduction with 80% power and α = 0.05 in a 4-year trial with annual assessments. The green line shows the sample size needed when only amyloid is used as an inclusion criterion and inter-network connectivity between the DMN and DAN is not considered. The orange line shows the required sample size when positive inter-network correlations between the DMN and DAN and amyloid are used as inclusion criteria. The blue line shows the required sample size when negative inter-network correlations between the DMN and DAN and amyloid are used as inclusion criteria

Power estimations were performed in the cognitively normal group to investigate the added value of adding functional inter-network connectivity to higher levels of Aβ burden as an inclusion criterion on the required sample size per arm in clinical trials (Fig. 3).

These results show that selecting cognitively normal participants with higher levels of Aβ burden and who also have a negative DMN-DAN correlation may greatly reduce the sample size needed to detect amyloid-related changes in memory decline in clinical trials as compared with only including preclinical AD individuals or those with positive correlations. For instance, the required sample size per arm to detect an effect size of 0.3 (30%) can be reduced by 88% when using both amyloid and negative inter-network correlations (*n* = 37) as inclusion criteria in clinical trials towards asymptomatic AD, as opposed to considering only amyloid (*n* = 318) in the inclusion process.

While functional inter-network connectivity is a relatively affordable and easy measure to collect in participants, this additional requirement can increase the rate of screen failures at inclusion.

## Discussion

This study provides evidence for a dose-response relationship between Aβ burden and inter-network connectivity on memory decline over 4 years in both healthy and patient populations. Previous studies suggested that negative correlations between task-positive and task-negative networks might have a beneficial effect on memory in young individuals [13, 14]. We now show that in older adults these associations depend on disease stage and the burden of Aβ. In cognitively normal older individuals, negative correlations were associated with memory decline, and this association became stronger in individuals with higher levels of Aβ. Conversely, in patients with MCI, the magnitude of Aβ burden predicted the rate of memory decline associated with positive inter-network correlations.

These results suggest that the direction of baseline inter-network connectivity can provide additional information when combined with baseline Aβ burden about the rate of expected memory decline and can add important information to the complexity of factors contributing to cognitive decline. Additionally, these findings can have prognostic implications when using functional connectivity as a potential biomarker and show the importance of including both the direction of inter-network connectivity and Aβ burden for projecting cognitive trajectories as outcome, especially in preclinical AD.

Interestingly, the associations we observed involved mainly the DMN-DAN correlations. The DMN is one of the most investigated networks and is among the first networks where Aβ accumulates significantly early in the disease [41]. It is therefore likely that other functional networks, especially networks tightly coupled to the DMN, are indirectly susceptible to Aβ-related alterations. Animal and electrophysiological studies have provided evidence that Aβ burden leads to synaptic dysfunction and network disorganization [42].

Widespread inter-network reorganization under the influence of Aβ have indeed been reported for the coupling between DMN and DAN [43], DMN and SN [19], and DMN and FPN [43], suggesting that topographical closeness to certain hubs, such as the association cortices of the DMN, may put networks at increased risk for Aβ toxicity. Our findings now show that functional implications of the interactions between large-scale brain networks and amyloid in relation to memory decline were mainly observed for the DMN-DAN correlations, with borderline significant associations for the DMN-SN correlations. This is in line with previous research suggesting that an optimal interaction between these specific networks is important for cognitive functioning [13, 14, 44]. The fact that we observed associations between greater positive inter-network correlations and better memory performance over time in clinically normal individuals suggests the presence of compensation mechanisms involving possible reconfiguration of large-scale networks [45, 46]. In the context of preclinical AD, this compensation may provide the architectural basis for maintaining optimal memory performance in old age [45, 47]. These compensation mechanisms may be moderated by cognitive reserve [46]. Individuals with preclinical AD may be able to compensate for AD pathology in the form of positive inter-network correlations. Furthermore, previous studies reported differential connectivity patterns for the anterior versus posterior DMN [48]. Increased within-network functional connectivity in the anterior DMN was associated with higher levels of amyloid pathology in preclinical AD [21], indicative of regional compensatory mechanisms. However, as the disease progresses, compensatory mechanisms may be dependent on cognitive reserve to stave off memory decline. Attenuated negative correlations between the DMN-DAN may signal impending neuronal breakdown resulting from Aβ toxicity in the prodromal phase, a finding consistent with previous work [46].

The DMN and DAN may operate in a negative correlation pattern to facilitate attention-related processes during cognitive tasks. The DAN is thought to coordinate cross-talk between brain networks, especially during memory tasks. The coordination between brain networks may become especially important in MCI, where medial temporal lobe structures become functionally isolated from other regions or networks. In MCI, a coordinated antiphase functional connection between the DMN and DAN may be fundamental in maintaining optimal levels of memory performance [46].

Finally, functional connectivity deteriorates as the disease progresses to more advanced stages [48]. This may partially explain the lack of connectivity-related findings in the AD group, as coherent connectivity patterns between networks may dissipate leaving no association between inter-network connectivity and cognition.

Our dose-response findings relating Aβ burden to memory decline, depending on functional connectivity status, showed different patterns between cognitively normal individuals and patients with MCI. This has important implications for early detection of individuals at risk of cognitive decline and the selection of preclinical and prodromal AD individuals for trials using a combination of functional connectivity and Aβ. Patients with MCI showed a dose-response relationship between Aβ and memory decline for positive DMN-DAN correlations. In clinically normal individuals, Aβ has a dose-response relationship with memory decline when DMN-DAN correlations are negative. Selecting preclinical AD individuals with positive DMN-DAN correlations could lead to the selection of individuals where individuals with higher levels of Aβ have similar practice effects as individuals with lower levels of Aβ burden. Thus, the direction of inter-network connectivity in combination with Aβ burden can have important implications for participant selection in clinical trials. This notion is further outlined by the results from our power analysis showing that the selection of cognitively normal individuals with elevated Aβ levels and negative inter-network correlations reduced the required sample size by 88% to slow down memory decline by 30%.

Furthermore, we did not observe independent or synergistic effects of inter-network connectivity or Aβ on cognition in patients with AD in the 2-year follow-up period. This could be related to widespread deposits of both Aβ and tau that may have impacted the integrity of the entire brain. We also did not observe between-group differences in inter-network connectivity of the DMN-DAN at baseline, which is in contrast to previous reports in similar populations [17–19]. These discrepancies may be related to sample size, methodological differences (regional versus network-based analyses), and the larger age range of the participants in our study.

Interestingly, the results found in our study seem to be domain-specific for memory. This may be explained by the DMN being specifically associated with memory functions [10, 46]. Since we found the most convincing results in the DMN-DAN correlation, this may emphasize the domain-specific nature of the interaction between DMN-DAN correlation, amyloid burden, and memory decline in preclinical AD.

### Limitations

Due to the observational nature of our study, our data do not allow for any causal inferences between changes in functional connectivity between networks or Aβ deposition. Determining the temporal direction of the three-way association between amyloid, memory decline, and inter-network functional connectivity requires further experimental investigation. Furthermore, longitudinal data of the AD group are limited to a follow-up period of 2 years. This may partially explain the lack of significant results found in this group, although it is also possible that the disease process and associated pathological accumulations are too widespread and may have affected functional networks in multiple ways, all associated with cognitive decline.

### Future directions

With the recent development of tau positron emission tomography tracers, future studies can investigate how tau pathology may impact inter-network connectivity since research has suggested a dynamic influence of tau on connectivity within networks, depending on Aβ levels [49–51]. Results from such a study could ultimately culminate in clinical trials where participants can be chosen using multi-modal selective criteria for memory decline due to AD.

Additionally, future studies should investigate whether carriers of the apolipoprotein ε4 allele show stonger synergistic effects between AD pathology [52, 53] and inter-network correlations on cognitive decline. Such investigations may further refine the selection criteria for trials and may have implications for determining response to treatment.

Finally, previous research has shown that AD variants, such as posterior cortical atrophy and early-onset AD, may have unique networks that are preferentially affected in the disease process [54]. Thus, future research may want to investigate how Aβ, inter-network correlations, and cognitive decline are associated in these different variants of AD and whether inter-network correlations may be associated with different cognitive domains in these variants.

## Conclusions

In conclusion, our results show that the direction of inter-network connectivity provides additional information to baseline Aβ burden about the rate of expected memory decline. These findings add important information for the understanding of factors contributing to cognitive decline. These results also suggest that when using functional connectivity as a biomarker or selection criterion for trials in preclinical populations, the directionality of inter-network connectivity might aid in selecting individuals that are more likely to be on an Aβ-related negative memory trajectory. Including information about both Aβ burden and inter-network functional connectivity can improve recruitment strategies and decrease the time to determine the efficacy of clinical trials in the asymptomatic phase of the disease.

## Additional file

Additional file 1: **Table S1.** List of participant IDs included in final analyses. **Table S2.** Peak coordinates of regions of interest in the MNI space. Box 1. Regression model construction. **Table S3.** Tukey tests for differences of baseline characteristics. **Table S4.** Summary of maximum motion parameter values and scrubbed volumes across groups. **Table S5.** Baseline results. **Table S6.** Associations between functional inter-network connectivity and memory decline in the whole sample. **Table S7.** Longitudinal linear effects of inter-network functional connectivity between each network pair on memory, per group. **Table S8.** Associations between group, inter-network functional connectivity, and decline in executive functions. **Table S9.** Associations between functional connectivity, time, amyloid status, memory, and executive functioning in the cognitively normal group. (DOCX 68 kb)

## Abbreviations

Aβ: Amyloid-beta
AD: Alzheimer’s disease
ADNI: Alzheimer’s Disease Neuroimaging Initiative
DAN: Dorsal attention network
DMN: Default mode network
FDR: False-discovery rate
FPN: Frontoparietal network
MCI: Mild cognitive impairment
ROI: Region of interest
SN: Salience network
