# Supplementary material for: Inter-network connectivity and amyloid-beta linked to cognitive decline in preclinical Alzheimer’s disease: a longitudinal cohort study
Source: Alzheimers Res Ther. 2018 Aug 28;10:88. doi: 10.1186/s13195-018-0420-9 (PMC6114059; doi:10.1186/s13195-018-0420-9)
Supplement: Supplementary file 1 — Table S1. List of participant IDs included in final analyses. Table S2. Peak coordinates of regions of interest in the MNI space. Box 1. Regression model construction. Table S3. Tukey tests for differences of baseline characteristics. Table S4. Summary of maximum motion parameter values and scrubbed volumes across groups. Table S5. Baseline results. Table S6. Associations between functional inter-network connectivity and memory decline in the whole sample. Table S7. Longitudinal linear effects of inter-network functional connectivity between each network pair on memory, per group. Table S8. Associations between group, inter-network functional connectivity, and decline in executive functions. Table S9. Associations between functional connectivity, time, amyloid status, memory, and executive functioning in the cognitively normal group. (DOCX 68 kb) [file 13195_2018_420_MOESM1_ESM.docx]

**Supplementary material**

**Inter-network connectivity and amyloid-β linked to cognitive decline in preclinical Alzheimer’s disease: a longitudinal cohort study**

# Roy W.E. van Hooren, MSc^1^; Joost M. Riphagen, MD, MSc^1,2^; Heidi I.L. Jacobs, PhD^1,3,4^; For the Alzheimer’s Disease Neuroimaging Initiative*

^1^Faculty of Health, Medicine and Life Sciences; School for Mental Health and Neuroscience, Department of Psychiatry and Neuropsychology, Alzheimer Center Limburg, Maastricht University, Maastricht, The Netherlands.

^2^Department of Anesthesiology, Sankt-Willibrord Spital, Emmerich, Germany

^3^Faculty of Psychology and Neuroscience, Department of Cognitive Neuroscience, Maastricht University, Maastricht, The Netherlands.

^4^Division of Nuclear Medicine and Molecular Imaging, Department of Radiology, Massachusetts General Hospital/Harvard Medical School, Boston, MA.

*Please refer to the authors’ contributions section on page 25.

Corresponding author: Roy W.E. van Hooren

Postal Address: Dr. Tanslaan 12, 6229 ET, Maastricht, the Netherlands

Phone: +31 6 24477702

Email: Roy W.E. van Hooren: roy.vanhooren@maastrichtuniversity.nl

Joost M. Riphagen: joost.riphagen@maastrichtuniversity.nl

Heidi I.L. Jacobs: h.jacobs@maastrichtuniversity.nl

# **Table S1. List of participant IDs included in final analyses**

| 031_S_4005 | 018_S_4349 | 013_S_4595 | 002_S_4251 | 006_S_4515 | 006_S_4867 |
| --- | --- | --- | --- | --- | --- |
| 031_S_4024 | 130_S_4352 | 018_S_4597 | 019_S_4252 | 136_S_4517 | 018_S_4868 |
| 012_S_4026 | 006_S_4357 | 130_S_4605 | 002_S_4262 | 002_S_4521 | 130_S_4883 |
| 031_S_4029 | 006_S_4363 | 013_S_4616 | 013_S_4268 | 130_S_4542 | 018_S_4889 |
| 031_S_4032 | 019_S_4367 | 130_S_4641 | 002_S_4270 | 006_S_4546 | 013_S_4917 |
| 031_S_4042 | 013_S_4395 | 012_S_4643 | 019_S_4285 | 019_S_4548 | 130_S_4925 |
| 012_S_4094 | 018_S_4399 | 002_S_4654 | 019_S_4293 | 019_S_4549 | 031_S_4947 |
| 012_S_4128 | 018_S_4400 | 130_S_4660 | 130_S_4294 | 100_S_4556 | 006_S_4960 |
| 031_S_4149 | 130_S_4405 | 053_S_4661 | 018_S_4313 | 053_S_4557 | 130_S_4971 |
| 006_S_4150 | 130_S_4415 | 006_S_4679 | 130_S_4343 | 053_S_4578 | 130_S_4982 |
| 006_S_4153 | 130_S_4417 | 019_S_4680 | 010_S_4345 | 013_S_4580 | 130_S_4984 |
| 002_S_4171 | 136_S_4433 | 018_S_4696 | 006_S_4346 | 031_S_4590 | 013_S_4985 |
| 012_S_4188 | 002_S_4447 | 006_S_4713 | 130_S_5059 | 019_S_5012 | 012_S_4987 |
| 006_S_4192 | 006_S_4449 | 031_S_4721 | 053_S_5070 | 002_S_5018 | 130_S_4990 |
| 031_S_4194 | 130_S_4468 | 130_S_4730 | 013_S_5071 | 130_S_5006 | 053_S_4813 |
| 031_S_4203 | 100_S_4469 | 002_S_4746 | 100_S_5106 | 130_S_4997 | |
| 002_S_4213 | 002_S_4473 | 013_S_4791 | 002_S_4229 | 006_S_4485 | |
| 031_S_4218 | 031_S_4474 | 002_S_4799 | 002_S_4237 | 031_S_4496 | |
| 002_S_4219 | 031_S_4476 | 018_S_4809 | 130_S_4250 | 100_S_4512 | |
| 002_S_4225 | 012_S_4849 | 019_S_4835 | 130_S_4817 | 019_S_4477 | |

# **Table S2. Peak coordinates of regions of interest in MNI space**

| **Region^a^** | **X** | **Y** | **Z** |
| --- | --- | --- | --- |
| **DMN** | | | |
| MPFC | 1 | 55 | -3 |
| LP (L) | -39 | -77 | 33 |
| LP (R) | 47 | -67 | 29 |
| PCC | 1 | -61 | 38 |
| **DAN** | | | |
| FEF (L) | -27 | -9 | 64 |
| FEF (R) | 30 | -6 | 64 |
| IPS (L) | -39 | -43 | 52 |
| IPS (R) | 39 | -42 | 54 |
| **SN** | | | |
| ACC | 0 | 22 | 35 |
| Insula (L) | -44 | 13 | 1 |
| Insula (R) | 47 | 14 | 0 |
| RPFC (L) | -32 | 45 | 27 |
| RPFC (R) | 32 | 46 | 27 |
| SMG (L) | -60 | -39 | 31 |
| SMG (R) | 62 | -35 | 32 |
| **FPN** | | | |
| LPFC (L) | -43 | 33 | 28 |
| LPFC (R) | 41 | 38 | 49 |
| PPC (L) | -46 | -58 | 30 |
| PPC (R) | 52 | -52 | 45 |

Abbreviations: ACC, Anterior Cingulate Cortex; DAN, Dorsal Attention Network; DMN, Default Mode Network; FEF, Frontal Eye Fields; FPN, Frontoparietal Network; IPS, Intraparietal Sulcus; LP, Lateral Parietal region; LPFC, Lateral Prefrontal Cortex; MPFC, Medial Prefrontal Cortex; PCC, Posterior Cingulate Cortex; PPC, Posterior Parietal Cortex; RPFC, Rostral Prefrontal Cortex; SMG, Supramarginal Gyrus; SN, Salience Network.

^a^Hemisphere of nodes is indicated between brackets; L = left hemisphere, R = right hemisphere.

**Supplementary Box 1. Regression model construction**

*Outcome_ij_= β_1_* + *β_2_Age_i_* + *β_3_Education_i_* + *β_4_Sex_i_* + *β_5_ICV_i_* + *β_6_ PredictorA_i_* + *β_7_ PredictorB_i_* + *β_8_Time_ij_* + *[β_9_ PredictorA_i_***Timeij]* + *[ β_10_PredictorB_i_***Time_ij_]* +

*[β_11_ PredictorA_i_***PredictorB_i_***Time_ij_]* + *[β_12_ PredictorA_i_***(Time_ij_^2^)]* +

*[β_13_PredictorB_i_***(Time_ij_^2^)]* + *[β_14_ PredictorA_i_***PredictorB_i_***(Time_ij_^2^)]* + *b_si_ Time_ij_* + *b_1i_* + *ε_i_*

Outcome: outcome variable measured over time (ADNI Memory composite);

Age_i_, Education_i_, Sex_i_, *ICV_i_*: age, education, sex or intracranial volume (ICV) for each participant;

Predictor A/B: independent variables of interest for the investigated model;

Time_ij_: time at testing session, relative to baseline testing session;

b_1i_: random intercept for each participant;

b_si_: random slope for each participant

ε_i_: error term for each participant

# **Table S3. Tukey tests for differences of baseline characteristics^a^**

| **Groups** | **Difference** | **95% CI Lower bound** | **95% CI Upper bound** | **Adjusted *p* value^b^** |
| --- | --- | --- | --- | --- |
| **AV45** | | | | |
| MCI- v CN | -0.14 | -0.25 | -0.03 | < 0.001 |
| MCI+ v CN | 0.24 | 0.13 | 0.34 | < 0.001 |
| AD v CN | 0.34 | 0.23 | 0.46 | < 0.001 |
| MCI+ v MCI- | 0.38 | 0.27 | 0.48 | < 0.001 |
| AD v MCI- | 0.49 | 0.37 | 0.60 | < 0.001 |
| AD v MCI+ | 0.11 | 0.00 | 0.21 | 0.05 |
| **ADNI-EF** | | | | |
| MCI- v CN | -0.55 | -1.07 | -0.03 | 0.03 |
| MCI+ v CN | -0.46 | -0.94 | 0.02 | 0.07 |
| AD v CN | -1.74 | -2.27 | -1.20 | < 0.001 |
| MCI+ v MCI- | 0.09 | -0.40 | 0.58 | 0.96 |
| AD v MCI- | -1.19 | -1.72 | -0.65 | < 0.001 |
| AD v MCI+ | -1.28 | -1.78 | -0.77 | < 0.001 |
| **ADNI-Mem** | | | | |
| MCI- v CN | -0.14 | -0.25 | -0.03 | 0.01 |
| MCI+ v CN | 0.24 | 0.13 | 0.34 | < 0.001 |
| AD v CN | 0.34 | 0.23 | 0.46 | < 0.001 |
| MCI+ v MCI- | 0.38 | 0.27 | 0.48 | < 0.001 |
| AD- v MCI- | 0.49 | 0.37 | 0.60 | < 0.001 |
| AD- v MCI+ | 0.11 | 0.00 | 0.21 | 0.05 |
| **CDR-SB** | | | | |
| MCI- v CN | 1.21 | 0.53 | 1.88 | < 0.001 |
| MCI+ v CN | 1.85 | 1.22 | 2.48 | < 0.001 |
| AD v CN | 4.36 | 3.67 | 5.06 | < 0.001 |
| MCI+ v MCI- | 0.64 | 0.01 | 1.28 | 0.046 |
| AD v MCI- | 3.16 | 2.46 | 3.86 | < 0.001 |
| AD v MCI+ | 2.51 | 1.86 | 3.17 | < 0.001 |
| **Follow-up time** | | | | |
| MCI- v CN | -0.33 | -0.76 | 0.10 | 0.19 |
| MCI+ v CN | -0.16 | -0.55 | 0.22 | 0.69 |
| AD v CN | -0.73 | -1.22 | -0.23 | 0.001 |
| MCI+ v MCI- | 0.17 | -0.24 | 0.58 | 0.72 |
| AD v MCI- | -0.39 | -0.91 | 0.12 | 0.20 |
| AD v MCI+ | -0.56 | -1.04 | -0.08 | 0.01 |

Abbreviations: AD: Alzheimer’s Disease; ADNI-EF: ADNI executive functions score composite; ADNI-Mem: ADNI memory score composite; APOE ε4, apolipoprotein ε4; AV45: ^18^F-AV-45 florbetapir; CDR-SB: Clinical Dementia Rating scale – Sum of Boxes; CI: Confidence interval; CN: Cognitively Normal; MCI: Mild Cognitive Impairment, - and + indicate amyloid negative- or positive grouping, respectively.

^a^Only characteristics showing a significant difference at baseline between any of the groups are listed here.

^b^*p* Values are adjusted with family-wise correction.

# **Table S4. Summary of maximum motion parameter values and scrubbed volumes across groups**

| **Parameter** | **CN** | **MCI-** | **MCI+** | **AD** | ***p* Value^a^** |
| --- | --- | --- | --- | --- | --- |
| Motion 1^b^ | 0.14 (0.08) | 0.18 (0.12) | 0.14 (0.09) | 0.13 (0.09) | 0.31 |
| Motion 2^b^ | 0.35 (0.29) | 0.34 (0.26) | 0.26 (0.13) | 0.31 (0.23) | 0.37 |
| Motion 3^b^ | 0.41 (0.65) | 0.32 (0.21) | 0.36 (0.48) | 0.39 (0.24) | 0.90 |
| Motion 4^b^ | 0.01 (< 0.01) | 0.01 (0.01) | < 0.01 (< 0.01) | < 0.01 (0.01) | 0.18 |
| Motion 5^b^ | < 0.01 (< 0.01) | < 0.01 (0.01) | < 0.01 (< 0.01) | < 0.01 (< 0.01) | 0.12 |
| Motion 6^b^ | < 0.01 (< 0.01) | < 0.01 (< 0.01) | < 0.01 (< 0.01) | < 0.01 (< 0.01) | 0.19 |
| Scrubbed volumes^c^ | 26.71 (23.70) | 27.37 (20.48) | 25.11 (20.30) | 24.30 (15.70) | 0.94 |

^a^*p* Values were calculated using ANOVA.

^b^The maximum displacement of each motion parameter was calculated for each participant. Group averages were calculated from these numbers and standard deviations are shown in brackets behind the average values. Motion parameter values are given in millimeters.

^c^Scrubbed volumes values are given in the average number of scrubbed volumes per perticipant in each group. Standard deviations are shown in brackets behind the averages. Scrubbed volumes are volumes that were regressed out due to a total displacement larger than 0.5mm.

**Table S5. Baseline results**

## **Associations between baseline inter-network functional connectivity and baseline memory performance and executive functions in the whole sample^a,b^**

| **Network** | **Estimate** | **SE** | **T Value** | ***p* Value** |
| --- | --- | --- | --- | --- |
| **Memory** | | | | |
| **DMN-DAN (n=115, number of observations = 115)** | | | | |
| DMN-DAN | 0.13 | 0.29 | 0.47 | 0.64 |
| **DMN-SN (n=115, number of observations = 115)** | | | | |
| DMN-SN | -0.16 | 0.30 | -0.54 | 0.59 |
| **DMN-FPN (n=115, number of observations = 115)** | | | | |
| DMN-FPN | 0.22 | 0.30 | 0.73 | 0.47 |
| **Executive functions** | | | | |
| **DMN-DAN (n=115, number of observations = 115)** | | | | |
| DMN-DAN | -0.08 | 0.30 | -0.28 | 0.78 |
| **DMN-SN (n=115, number of observations = 115)** | | | | |
| DMN-SN | 2.53 | 0.31 | -0.81 | 0.42 |
| **DMN-FPN (n=115, number of observations = 115)** | | | | |
| DMN-FPN | 0.67 | 0.31 | 2.15 | 0.03 |

## **Associations between inter-network functional connectivity on baseline memory and executive functions performance for each group separately^a^**

| **Network** | **Estimate** | **SE** | **T Value** | ***p* Value** |
| --- | --- | --- | --- | --- |
| **Memory** | | | | |
| **CN (n=28, number of observations = 28)** | | | | |
| DMN-DAN | -0.39 | 0.33 | -1.21 | 0.24 |
| DMN-SN | -0.48 | 0.32 | -1.51 | 0.15 |
| DMN-FPN | -0.37 | 0.41 | -0.92 | 0.37 |
| **MCI- (n=27, number of observations = 27)** | | | | |
| DMN-DAN | 0.04 | 0.33 | 0.12 | 0.90 |
| DMN-SN | -0.10 | 0.41 | -0.24 | 0.81 |
| DMN-FPN | -0.44 | 0.35 | -1.27 | 0.22 |
| **MCI+ (n=36, number of observations = 36)** | | | | |
| DMN-DAN | 0.07 | 0.39 | 0.18 | 0.86 |
| DMN-SN | -0.07 | 0.34 | -0.20 | 0.84 |
| DMN-FPN | -0.13 | 0.42 | -0.30 | 0.77 |
| **AD (n=24, number of observations = 24)** | | | | |
| DMN-DAN | -0.18 | 0.47 | -0.39 | 0.70 |
| DMN-SN | -0.59 | 0.47 | -1.26 | 0.22 |
| DMN-FPN | -0.31 | 0.43 | -0.71 | 0.49 |
| **Executive functions** | | | | |
| **CN (n=28, number of observations = 28)** | | | | |
| DMN-DAN | -0.48 | 0.43 | -1.13 | 0.27 |
| DMN-SN | 0.08 | 0.44 | 0.18 | 0.86 |
| DMN-FPN | 0.37 | 0.54 | 0.70 | 0.49 |
| **MCI- (n=27, number of observations = 27)** | | | | |
| DMN-DAN | -0.05 | 0.47 | -0.12 | 0.91 |
| DMN-SN | -0.31 | 0.58 | -0.54 | 0.60 |
| DMN-FPN | 1.25 | 0.44 | 2.83 | 0.01 |
| **MCI+ (n=36, number of observations = 36)** | | | | |
| DMN-DAN | -0.37 | 0.52 | -0.71 | 0.48 |
| DMN-SN | 0.23 | 0.47 | 0.48 | 0.63 |
| DMN-FPN | -0.49 | 0.57 | -0.85 | 0.40 |
| **AD (n=24, number of observations = 24)** | | | | |
| DMN-DAN | -0.20 | 0.51 | -0.39 | 0.70 |
| DMN-SN | 0.18 | 0.53 | 0.34 | 0.74 |
| DMN-FPN | 0.11 | 0.48 | 0.23 | 0.82 |

Abbreviations: AD: Alzheimer’s Disease; DAN: Dorsal Attention Network; DMN: Default Mode Network; FPN: Frontoparietal Network; MCI: Mild Cognitive Impairment, - and + indicate amyloid negative- or positive grouping, respectively; SE: Standard Error; SN: Salience Network.

^a^ Regression models are adjusted for age, intracranial volume, sex and years of education. Beta coefficients are unstandardized.

^b^ Results are obtained using CN as the reference group.

**Table S6. Associations between functional inter-network connectivity and memory decline in the whole sample**

| **Fitted model** | **Est.** | **SE** | **95% CI** | **DF** | **T Value** | ***p* Value** |
| --- | --- | --- | --- | --- | --- | --- |
| **Whole sample (n = 115, number of observations = 433)** | | | | | | |
| Time*DMN-DAN | -0.04 | 0.09 | -0.22 to 0.15 | 316 | -0.40 | 0.69 |
| Time*DMN-SN | 0.06 | 0.09 | -0.12 to 0.23 | 316 | 0.65 | 0.52 |
| Time*DMN-FPN | 0.04 | 0.10 | -0.16 to 0.24 | 316 | 0.39 | 0.70 |

Abbreviations: CI: Confidence interval; DAN: Dorsal Attention Network; DF, Degrees of Freedom; DMN: Default Mode Network; FPN: Frontoparietal Network; SE: Standard Error; SN: Salience Network.

# **Table S7. Longitudinal linear effects of inter-network functional connectivity between each network pair on memory, per group^a^**

| **Fitted model** | **Estimate** | **SE** | **95 % CI** | **DF** | **T Value** | ***p* Value** |
| --- | --- | --- | --- | --- | --- | --- |
| **CN (n=28, number of observations = 118)** | | | | | | |
| DMN-DAN*Time | 0.37 | 0.11 | 0.16 to 0.58 | 88 | 3.49 | 0.001 |
| DMN-SN*Time | 0.39 | 0.10 | 0.20 to 0.59 | 88 | 4.06 | < 0.001 |
| DMN-FPN*Time | 0.25 | 0.14 | -0.02 to 0.52 | 88 | 1.87 | 0.06 |
| **MCI- (n=27, number of observations = 100)** | | | | | | |
| DMN-DAN*Time | -0.10 | 0.12 | -0.33 to 0.13 | 71 | -0.85 | 0.40 |
| DMN-SN*Time | 0.11 | 0.12 | -0.13 to 0.35 | 71 | 0.88 | 0.38 |
| DMN-FPN*Time | 0.08 | 0.14 | -0.19 to 0.35 | 71 | 0.58 | 0.56 |
| **MCI+ (n=36, number of observations = 146)** | | | | | | |
| DMN-DAN*Time | -0.49 | 0.11 | -0.72 to -0.26 | 108 | -4.27 | < 0.001 |
| DMN-SN*Time | -0.28 | 0.09 | -0.46 to -0.10 | 108 | -3.09 | 0.001 |
| DMN-FPN*Time | 0.21 | 0.13 | -0.04 to 0.46 | 108 | 1.66 | 0.10 |
| **AD (n=24, number of observations = 69)** | | | | | | |
| DMN-DAN*Time | 0.10 | 0.19 | -0.28 to 0.49 | 43 | 0.53 | 0.60 |
| DMN-SN*Time | -0.03 | 0.20 | -0.44 to 0.37 | 43 | -0.16 | 0.87 |
| DMN-FPN*Time | -0.27 | 0.18 | -0.63 to 0.08 | 43 | -1.54 | 0.13 |

Abbreviations: AD: Alzheimer’s Disease; CN: Cognitively Normal; DAN: Dorsal Attention Network; DF: Degrees of Freedom; DMN: Default Mode Network; FPN: Frontoparietal Network; MCI: Mild Cognitive Impairment, - and + indicate amyloid negative- or positive state, respectively; SE: Standard Error; SN: Salience Network.

^a^ Regression models are adjusted for age, intracranial volume, sex and education. Beta coefficients are unstandardized.

**Table S8. Associations between group, inter-network functional connectivity and decline in executive functions^a,b^**

| **Fitted model** | **Est.** | **SE** | **95% CI** | **DF** | **T Value** | ***p* Value** |
| --- | --- | --- | --- | --- | --- | --- |
| **DMN-DAN (n = 115, number of observations = 433)** | | | | | | |
| Time*MCI-*DMN-DAN | 0.17 | 0.26 | -0.35 to 0.69 | 310 | 0.64 | 0.52 |
| Time*MCI+*DMN-DAN | -0.18 | 0.28 | -0.73 to 0.38 | 310 | -0.63 | 0.53 |
| Time*AD*DMN-DAN | -0.02 | 0.36 | -0.74 to 0.70 | 310 | -0.05 | 0.96 |
| **DMN-SN (n = 115, number of observations = 433)** | | | | | | |
| Time*MCI-*DMN-SN | 0.16 | 0.28 | -0.38 to 0.71 | 310 | 0.59 | 0.55 |
| Time*MCI+*DMN-SN | 0.03 | 0.24 | -0.44 to 0.50 | 310 | 0.11 | 0.91 |
| Time*AD*DMN-SN | -0.19 | 0.37 | -0.92 to 0.54 | 310 | -0.52 | 0.61 |
| **DMN-FPN (n = 115, number of observations = 433)** | | | | | | |
| Time*MCI-*DMN-FPN | 310 | 0.69 | -0.39 to 0.82 | 310 | 0.69 | 0.49 |
| Time*MCI+*DMN-FPN | 310 | 0.23 | -0.51 to 0.65 | 310 | 0.23 | 0.82 |
| Time*AD*DMN-FPN | 310 | -1.14 | -1.15 to 0.31 | 310 | -1.14 | 0.26 |

Abbreviations: AD, Alzheimer’s Disease; CI, Confidence Interval; DAN, Dorsal Attention Network; DF, Degrees of Freedom; DMN, Default Mode Network; FDR, False Discovery Rate; FPN, Frontoparietal Network; MCI, Mild Cognitive Impairment, - and + indicate amyloid-β negative- or positive grouping, respectively; SE, Standard Error; SN, Salience Network.

^a^Results are acquired using the cognitively normal group as reference group.

^b^Regression models are adjusted for age, intracranial volume, sex and education. Beta coefficients are unstandardized.

**Table S9. Associations between functional connectivity, time, amyloid status, memory and executive functioning in the cognitively normal group**

| **Fitted model** | **Est.** | **SE** | **95% CI** | **DF** | **T Value** | ***p* Value** |
| --- | --- | --- | --- | --- | --- | --- |
| **Memory (n=28, number of observations = 118 )** | | | | | | |
| Time*Amyloid status*DMN-DAN | 0.61 | 0.30 | 0.01 to 1.20 | 86 | 2.02 | 0.046 |
| Time*Amyloid status*DMN-SN | 0.53 | 0.31 | -0.10 to 1.15 | 86 | 1.68 | 0.10 |
| Time*Amyloid status*DMN-FPN | 0.29 | 0.53 | -0.76 to 1.34 | 86 | 0.55 | 0.58 |
| **Executive functioning** **(n=28, number of observations = 118 )** | | | | | | |
| Time*Amyloid status*DMN-DAN | 0.38 | 0.37 | -0.35 to 1.11 | 86 | 1.03 | 0.31 |
| Time*Amyloid status*DMN-SN | -0.09 | 0.38 | -0.85 to 0.68 | 86 | -0.22 | 0.82 |
| Time*Amyloid status*DMN-FPN | -0.81 | 0.56 | -1.92 to 0.30 | 86 | -1.45 | 0.15 |

Abbreviations: CI: Confidence interval; DAN: Dorsal Attention Network; DF, Degrees of Freedom; DMN: Default Mode Network; FPN: Frontoparietal Network; SE: Standard Error; SN: Salience Network.
